# The Impact of Dehydration and Hyperthermia on Circulatory Glutathione Metabolism after Exercise in the Heat with Insights into the Role of Erythrocytes

**DOI:** 10.3390/life11111144

**Published:** 2021-10-27

**Authors:** Denise de Melo-Marins, Juliano Boufleur Farinha, Franccesco Pinto Boeno, Alexandra Ferreira Vieira, Samuel Vargas Munhoz, Gabriela Cristina dos Santos, Mauricio Krause, Orlando Laitano, Alvaro Reischak-Oliveira

**Affiliations:** 1 School of Physical Education, Physical Therapy and Dance, Federal University of Rio Grande do Sul (UFRGS), Porto Alegre 90040-060, Brazil; juliano.farinha@ebserh.gov.br (J.B.F.); boeno.f@ufl.edu (F.P.B.); alexandra.vieira@ufrgs.br (A.F.V.); 00309850@ufrgs.br (S.V.M.); 00243261@ufrgs.br (G.C.d.S.); 00009478@ufrgs.br (A.R.-O.); 2 Laboratory of Inflammation, Metabolism and Exercise Research (LAPIMEX), Laboratory of Cellular Physiology, Department of Physiology, Institute of Basic Health Sciences, Federal University of Rio Grande do Sul, Porto Alegre 90040-060, Brazil; mauricio.krause@ufrgs.br; 3 Department of Nutrition and Integrative Physiology, Florida State University, Tallahassee, FL 32306, USA; olaitano@fsu.edu

**Keywords:** oxidative stress, heat, hydration, exercise

## Abstract

Background: Reduced glutathione (GSH) is one of the main thiols involved in antioxidant defense. Changes in circulatory levels of GSH during exercise are associated with hyperthermia and dehydration. The mechanisms by which these alterations occur are not entirely known. We hypothesize that erythrocytes could be an important source of circulatory GSH during heat stress conditions. We performed two separate experiments to address this hypothesis. Methods: In the first experiment, we sought to investigate the impact of exercise in the heat and dehydration on erythrocyte levels of GSH. A total of 10 men performed 60 min of cycling at 60% VO_2_peak in the heat (38.0 ± 0.9 °C) or in a control temperate environment (23.0 ± 1.0 °C), both with and without dehydration. Relative humidity ranged from 50 to 70%. Blood samples were taken before and after exercise to measure GSH and oxidized (GSSG) glutathione. In the second experiment, erythrocytes were isolated from blood samples taken at rest and heated in vitro to determine the impact of heat on erythrocyte glutathione content. Tubes with erythrocytes were exposed to water baths at different temperatures; one tube was exposed to a water bath at 35 °C and the other tube to a water bath at 41 °C for a period of 30 min. After exposure to heat, plasma and erythrocytes were extracted for GSH and GSSG analyses. Results: Dehydration decreased circulatory GSH, regardless of ambient temperature (temperate and heat decreased 15.35% and 30.31%, respectively), resulting in an altered redox balance. Heat increased GSH levels in vitro. Conclusion: Our data suggest that dehydration decreases circulatory GSH levels regardless of environmental temperature. In addition, in vitro data suggests that erythrocytes may contribute to the release of GSH during exposure to heat stress.

## 1. Introduction

Acute physical exercise induces the formation of reactive oxygen species (ROS) [1,2], which impairs the maintenance of cellular redox balance, promoting a transitory state of oxidative stress [3,4]. Exercise performed in the heat is associated with hyperthermia and dehydration, which further contributes to the formation of ROS, heightening the imbalance in the redox status [5,6,7,8,9]. Nevertheless, it remains unclear whether the effects of hyperthermia and dehydration on circulatory changes are direct or indirect.

Glutathione (GSH) is a thiol commonly reported in the exercise heat stress literature. GSH metabolism plays an important role as a buffer for the cellular redox state in skeletal muscle due to its water-soluble antioxidant properties [10]. GSH is formed by a tripeptide consisting of glutamate, cysteine and glycine, with the thiol group of cysteine being the active site responsible for its biochemical properties [11]. The role of GSH within the antioxidant defense involves its ability to react with ROS by donating hydrogen, as well as serving as a substrate for the action of the enzyme glutathione peroxidase (GPx), neutralizing the potential effects of ROS [11,12].

Circulatory changes in GSH content may reflect the response of erythrocytes to osmotic stress induced by hyperthermia and dehydration. Erythrocytes are continually exposed to high concentrations of oxygen and iron in hemoglobin, and these factors make these cells sensitive to oxidative damage [13], which, in turn, makes them a potential model for studying oxidative stress [14]. Studies in humans exploring the behavior of GSH during exercise measured the circulating levels of GSH [8,15,16]. Although hepatic cells are responsible for most GSH release, it has been suggested that skeletal muscles may also be a relevant source [8]. Nevertheless, it is also likely that GSH measured in the circulation may be influenced by erythrocyte sources during exercise in heat stress [15,16].

Changes in GSH levels in response to situations in which redox imbalance is present demonstrate its importance as an antioxidant defense mechanism [15,16,17,18,19]. Acute and chronic physical exercise promote changes in cellular and circulatory levels of GSH [2,20]. In addition, hyperthermia and dehydration have been shown to promote changes in GSH metabolism [5,8,16,21]. However, it is difficult to isolate the effects of dehydration from the effects of hyperthermia on GSH. Some studies demonstrate that dehydration increases post-exercise oxidized glutathione (GSSG) [5] as well as the greater release of GSH in the blood during exercise [8].

Therefore, the aim of this study was to investigate the effects of exercise in heat with and without dehydration and isolated heat stress in erythrocytes in vitro. We hypothesize that erythrocytes may be a source of GSH during heat stress conditions.

## 2. Materials and Method

### 2.1. Ethical Compliance and Participants

The present study’s protocol was approved by the Research Ethics Committee of the Federal University of Rio Grande do Sul under the number 79281817.2.0000.5347. All participants gave written informed consent, and procedures conformed to the code of Ethics of the Medical Association (Declaration of Helsinki). Ten healthy physically active men were recruited to the study. Table 1 describes the physical characteristics and functional status of the participants. All participants were physically active at a frequency of at least 3 times a week, getting the minimum recommended exercise time of ≥150 min per week. Participants took part in two separate experiments.

Experiment 1: This experiment was performed to determine the circulatory levels of GSH and GSSG during exercise in the heat with and without dehydration. Participants visited the laboratory on five separate occasions, with visits consisting of a preliminary visit and four experimental exercise sessions randomized and separated by at least four and at most seven days.

#### 2.1.1. Preliminary Visit

During the first visit, body composition was assessed via dual energy X-ray absorptiometry (DXA, Lunar Prodigy Primo, GE Healthcare, Chicago, IL, USA). To ensure euhydration status during assessment of body composition and throughout the test, participants were asked to consume 5 mL of water per kg of body weight ~1 h before that preliminary visit. Thereafter, participants performed an incremental cycling (ERGO-FIT, Pirmasens, Germany) test to exhaustion to determine VO_2_peak (Quark CPET, Cosmed, Rome, Italy). Initial workload was set at 30 W with increments of 30 W/min and a constant cadence of 70–75 rpm until exhaustion. The test was performed under a temperate environmental temperature (25 °C and ~55% relative humidity). VO_2_peak was used to set the exercise intensity of the subsequent exercise visits. In addition, participants were instructed to record their 24 h food intake and were asked to repeat the same diet pattern before the following visits.

#### 2.1.2. Experimental Visits 

The four experimental visits consisted of 60 min of cycling at 60% of VO_2_peak under different environmental conditions and hydration status. The visits were carried out either in the heat (H) (38.0 ± 0.9 °C) with (DE-H) or without (EU-H) dehydration, or in a thermoneutral environment (T) (23.0 ± 1.0 °C) with (DE-T) and without (EU-T) dehydration. Exercise sessions in the heat were carried out in an environmental chamber (Russells, The Netherlands). The relative humidity during the sessions ranged from 50 to 70%. Participants were advised not to perform physical exercises in the 24 h prior to the experimental visits. None of the participants were taking any antioxidant supplements and were asked to avoid drastic changes in regular exercise habits throughout study. Hydration levels were determined before the exercise sessions. For this, the following protocols were followed: (1) in the euhydration condition, participants were instructed to consume 5 mL of water/kg of body weight ~1 h before the visit; (2) in the dehydration condition, participants were instructed not to drink fluids during the 10 h preceding the experimental visit. The determination of hydration level prior to the participants’ exercise was determined by urine specific gravity (USG). USG values < 1.025 were considered to indicate euhydration status [22]. Upon arrival, participants emptied their bladder, and the urine sample was used to determine the USG. Pre-exercise body mass was recorded to estimate body fluid loss during the experimental conditions. Thereafter, the first blood sample was collected (5 mL) from the antecubital region while participants were comfortably seated. Then, participants were instructed to perform self-insertion (10 cm beyond the anal sphincter) of the rectal thermometer (Physitemp, Clifton, NJ, USA). Fluid consumption was not allowed during the sessions of 60 min of cycling at 60% of VO_2_peak. Immediately after exercise, a new blood collection was performed, followed by a urine collection and recording of post-exercise body mass. The pre- and post-exercise blood collection was used to determine hemoglobin, hematocrit, and for the analysis of GSH and GSSG. During exercise, heart rate (Polar, S610, Polar Electro Oy, Kempele, Finland) and rating of perceived exertion [23] were continuously recorded. 

#### 2.1.3. Blood Preparation and Assays

Blood samples (5 mL) were collected, and 1 mL was separated for immediate hemoglobin and hematocrit analyses. The remaining volume was stored in EDTA tubes. Hemoglobin was determined using a commercially available assay kit (Labtest, Lagoa Santa, Brazil). The hematocrit was determined in quadruplicate by microcentrifugation. Changes in plasma volume were estimated (Δ%) using hemoglobin and hematocrit values [24]. The tube containing 4 mL of whole blood was centrifuged at 454× *g* for 10 min at 4 °C, and 400 µL of the precipitate was separated. To deproteinize and lyse the cells, we added 1600 µL of sulfosalicylic acid (5%) and a new centrifugation was carried out at 1260× *g* for 10 min at 4 °C. After, 630 µL of the precipitate was separated and 70 µL of H_2_O was added. Then, the tube with the preparation was immediately frozen. The concentrations of GSH and GSSG were measured using a spectrophotometric kit (Sigma-Aldrich, St. Louis, MO, USA); both were measured at 412 nm in a microplate reader (Multiskan Go; Thermo Scientific, Waltham, MA, USA). This kit (Sigma-Aldrich, USA) contains the reagent for GSH masking. In brief, the reaction of dithiodinitrobenzoic acid with a thiol compound such as GSH produces a yellow compound. Regarding GSSG, it can be selectively determined by measuring the absorption derived from a colorimetric reaction of (5,5′-dithiobis (2-nitrobenzoic acid)) (DTNB) coupled with the enzymatic recycling system (YANG et al., 2020). GSH and GSSG levels were determined from comparisons with a linear GSH or GSSG standard curve, respectively. Lastly, the GSH/GSSG ratio was calculated.

Experiment 2: In the second experiment, performed on a separate visit, erythrocytes were heated in vitro. Eight subjects from experiment 1 participated in this experiment. The protocol consisted of a blood collection at rest to determine the contribution of the erythrocytes to the release of GSH in response to heat exposure.

#### 2.1.4. Blood Preparation, Heating, and Assays

Participants were previously euhydrated (they ingested 5 mL of water/kg of body mass ~1 h before the visit) and were instructed to avoid vigorous exercises in the previous 24 h. Blood (20 mL) was collected from the antecubital region at rest, and stored in tubes containing EDTA anticoagulant. Aliquots were set aside for immediate analysis of hemoglobin by colorimetry as described in experiment 1 and used later for the correction of GSH and GSSG values. Two 10 mL tubes were exposed to water baths set at different temperatures; one tube was exposed to a water bath set at 35 °C and the other was exposed to a water bath set at 41 °C for a period of 30 min. After exposure to heat, the tubes were centrifuged at 202× *g* for 10 min at 20 °C; plasma and erythrocytes were extracted, and immediately frozen. GSH and GSSG levels were measured as explained above for experiment 1 (Sigma-Aldrich, St. Louis, MO, USA).

### 2.2. Statistical Analysis

All data are represented as means ± SD. Normality of data was first assessed using the Shapiro-Wilk test and the homoscedasticity of the variances was assessed through the Levene test. Mixed model analysis of variance (ANOVA) was used to analyze the data of the experiment in humans using the Tukey post hoc test. For the analysis of the experiment 2 data (in vitro), paired *t*-tests were used for the analysis of parametric data, and Wilcoxon signed rank test was used for the non-parametric data. For the purpose of hypothesis testing, the 95% level of confidence was predetermined as the minimum criterion to denote a statistical difference (*p* < 0.05) and the effect size was calculated using Cohen’s d. All data analyses were performed using SPSS 20.0 for Windows (SPSS Inc., Chicago, IL, USA).

## 3. Results

### 3.1. Experiment 1

#### 3.1.1. Body Temperature, Hydration Status and Heart Rate 

**Figure 1:**
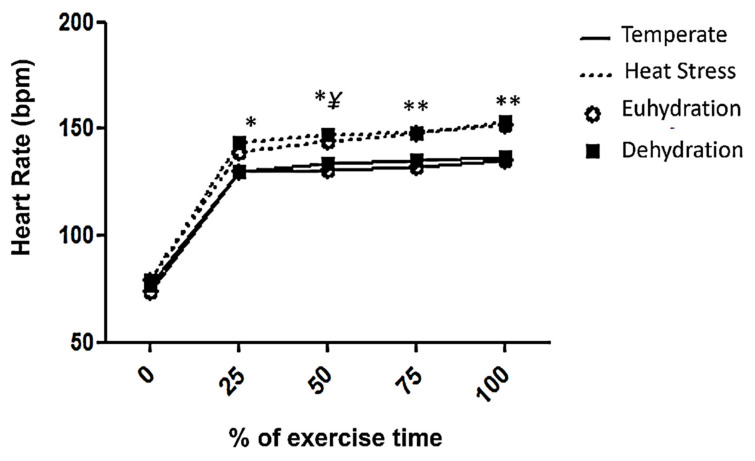
Heart rate response during exercise in the four experimental conditions. Data are represented as means ± SD (*n* = 10). T: temperate; H: heat stress; EU: euhydration; DE: dehydration. * DE-H significantly different from EU-T and HY-T (*p* < 0.05). ^¥^ EU-H significantly different from EU-T (*p* < 0.05). ** DE-H and EU-H significantly different from EU-T and DE-T (*p* < 0.05).

Body temperature increased in all experimental conditions when compared to rest (Table 2). The EU-H and DE-H conditions and the rectal temperature increases were 1 °C and 1.2 °C, respectively. Similar changes were also observed in body mass among conditions, with a significant decrease in body mass after exercise (Table 2). The percentage of dehydration and Hb increased after exercise in all conditions (Table 2). However, there was only an increase in Htc in exercise conditions in the hot environment. Exercising in the heat promoted an increase in heart rate after 30 min of exercise when compared to exercise in a temperate environment, regardless of the previous level of dehydration (Figure 1).

#### 3.1.2. Effects of Exercise, Heat, and Dehydration on Circulating Glutathione

**Figure 2:**
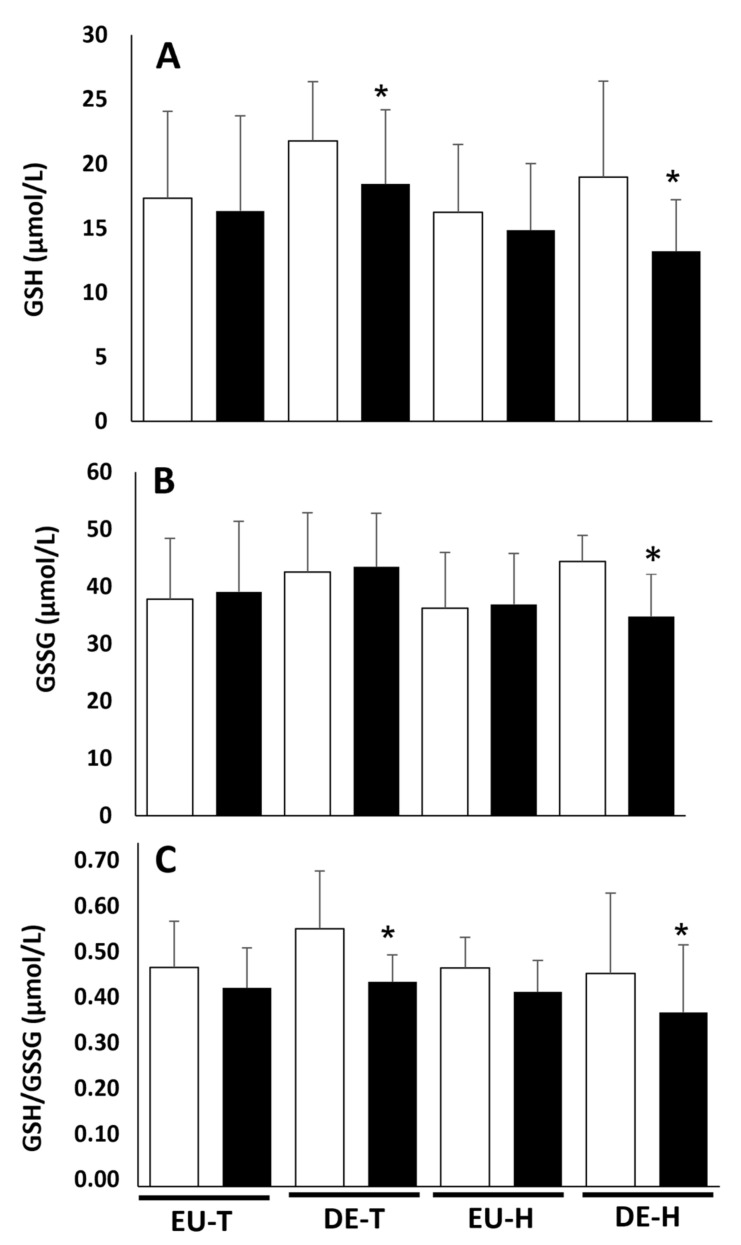
Effects of exercise performed in heat and in a temperate environment with or without dehydration on erythrocytes’ reduced glutathione (**A**), oxidized glutathione (**B**) and the GSH/GSSG ratio (**C**) at rest and after exercise. Data are represented as means ± SD (*n* = 8). T: temperate; H: heat stress; EU: euhydration; DE: dehydration. * Significantly different from rest (*p* < 0.05).

The EU-T condition did not change GSH and GSSG concentrations, which consequently did not change the GSH/GSSG ratio (Figure 2). On the other hand, the DE-T condition decreased GSH levels (*d* = 0.3) and the GSH/GSSG ratio (*d* = 0.48) (Figure 2). The EU-H condition did not change any of the variables. However, for the DE-H condition, reductions in GSH and GSSG concentrations were observed (respectively, *d* = 0.43, 0.62). The GSH/GSSG ratio also decreased in the DE-H condition (*d* = 0.26) (Figure 2).

### 3.2. Experiment 2

#### Effects of Heat Stress on Erythrocytes on Glutathione Release

No differences were observed in plasma GSH, GSSG or the GSH/GSSG ratio when compared to heating the blood to 35 °C and 41 °C (Table 3). Regarding the analysis of erythrocytes, there was an increase in GSH when these cells were heated to 41 °C compared to heating to 35 °C (*d* = −0.45) (Table 3). On the other hand, there were no changes regarding the GSSG and the GSH/GSSG ratio.

## 4. Discussion

The main finding of our study is that exercise performed in a state of dehydration (~2%), regardless of ambient temperature, decreases GSH and consequently the GSH/GSSG ratio. In addition, we demonstrate that hyperthermia, in vitro, increases the release of GSH by erythrocytes, likely contributing to the circulatory pool in conditions of heat stress. To our knowledge, this is the first study that directly investigated the release of GSH by erythrocytes during isolated heat stress in humans.

Hyperthermia and dehydration are considered important physiological stressors during exercise [25]. Their combined and isolated effects on GSH metabolism have been previously investigated. Hillman et al. (2011) demonstrated that exercise performed in a ~3% dehydration state resulted in a 29% increase in plasma GSSG regardless of ambient temperature. On the other hand, when exercise was performed in a euhydration state, this increase was attenuated [5]. In the present study, there was a decrease in GSSG when heat was associated with dehydration, which was accompanied by a decrease in GSH. These changes decreased the GSH/GSSG ratio, which is consistent with a state of redox imbalance. Similarly, we observed a decrease in GSH and the GSH/GSSG ratio in the thermoneutral and dehydration conditions. These findings suggest that dehydration, regardless of the ambient temperature that the exercise is performed in, promotes redox imbalance due to a decrease in GSH and, consequently, in the GSH/GSSG ratio. The studies by Laitano et al. (2010, 2012) and Hillman et al. (2011, 2013) demonstrated similar results, emphasizing that dehydration may be the main factor leading to oxidative stress.

The dehydration levels achieved in the present study are considered mild dehydration (2%). Studies demonstrating increases in circulatory levels of GSSG during exercise reached dehydration levels greater than 3%. This higher percentage of dehydration promotes changes in plasma volume that can modify circulating GSSG values. In our study, no differences were observed in plasma volume, which may also help explain the lack of changes in circulatory levels of GSSG.

Besides the effects of dehydration on glutathione metabolism, the effect of exercise under these conditions of dehydration and heat on circulatory levels of GSH has been investigated. For instance, dehydration of 3.5% body mass loss promoted the release of GSH into the circulation during moderate-intensity exercise [8]. In addition, moderate-intensity exercise in the heat without dehydration promoted the release of GSH into the bloodstream [16]. Our current results, which indicate that exercise performed in the heat without dehydration does not alter the redox balance, are in agreement with these previous studies.

The type of exercise may be an important factor influencing the responses of circulatory levels of glutathione during conditions of heat and dehydration. The type of exercise we used in this study differs from that used by previous investigations [8,16]. These studies used the single-legged knee extension exercise. In the present study, simultaneous muscular action of the lower limbs (cycling) was used. Although exercise intensity was moderate, changes in GSH levels can be directly or indirectly influenced by the type of exercise, muscle action and even by the way hyperthermia is induced (by exercising in the heat or just passively). Although it may not be possible to determine the behavior of GSH levels in these situations, it is well known that different tissues can contribute to these changes in GSH concentrations; therefore, it is important to highlight these differences when interpreting these findings.

Different physiological tissues have been proposed to release GSH into the bloodstream during exercise. Results from more classical studies have shown that there are decreases in the levels of GSH in the liver of animals during exercise [26,27,28], and that there are increases of GSH in plasma and skeletal muscle. Since the majority of human studies to date have investigated only circulatory levels of GSH, it is still unclear which sources contribute the most to GSH release during exercise, specifically during exercise in heat and in the presence of dehydration. By contrasting our in vivo and in vitro findings, it is possible to conjecture that the core temperature elicited by the participants in our in vivo trial may not have been sufficient to stimulate major GSH release by erythrocytes, at least when participants were euhydrated. The heat dose-response of GSH release by erythrocytes has yet to be determined in future experiments.

We observed that erythrocytes (in vitro) respond to the heat (41 °C) by increasing GSH levels. The exact mechanisms by which GSH metabolism is regulated at the level of erythrocytes are not entirely clear but seem to involve multidrug resistance protein 1 (MRP1). MRP1 regulates the levels of GSH in erythrocytes, and there is also (partially) an efflux of GSSG by this pump, which affects the levels of GSH in erythrocytes [29]. Previous work has shown that MRP1 expression and activity is a determinant of GSH metabolism in skeletal muscle and heart tissue after exercise [20]. Interestingly, hyperthermia can induce increased MRP1 expression in certain types of cells, collaborating with GSH/GSSG release [30]. Therefore, it appears that there is indeed a contribution of erythrocytes to the release of GSH, and that this also occurs during exposure to heat. Besides elucidating part of the antioxidant responses associated with moderate exercise in the heat, future research should investigate other sources of GSH release during exercise with heat stress. In addition, further research should analyze the activity of enzymes that regulate GSH levels during exposure of erythrocytes to heat, as well as the activity of the multidrug resistance-associated protein 1 (MRP1), correlating its activity with the GSH values in these cells.

## 5. Limitations

Our study is not without limitations. For instance, we did not analyze the enzymatic levels of glutathione reductase and peroxidase. The activities of these enzymes could better clarify the results found in GSH levels in erythrocytes after heat stress. More studies are warranted to elucidate the sources of GSH release during exercise, especially in situations of heat stress and dehydration. The temperature we stimulated the blood with was likely higher than the blood temperature (not measured) during the in vivo experiment. Studies measuring and matching temperature are warranted to determine the true contribution of erythrocytes to the circulatory pool of GSH in vivo.

## 6. Conclusions

In conclusion, the present study demonstrates, for the first time in humans, that dehydration is an important stimulus promoting redox imbalance during moderate exercise. Heat stress is an important stimulus for the release of GSH by erythrocytes, which could be a relevant source of GSH in human circulation during exercise in heat stress conditions.

## Figures and Tables

**Table 1:** Physical characteristics of subjects (*n* = 10).

Characteristic	Mean ± SD
Age (years)	26 ± 3.7
Height (cm)	177 ± 0.1
Body mass (kg)	79.7 ± 8.8
Body fat (%)	22.3 ± 4.5
BMI (kg/m^2^)	25.5 ± 2.6
VO_2_peak (mL/kg/min)	40.81 ± 4.7
Peak work rate (W)	288 ± 40.5

BMI: Body mass index.

**Table 2:** Changes in urine-specific gravity (USG), hemoglobin (Hb), hematocrit (Htc), plasma volume (PV), total body mass (BM), sweat rate (SR), dehydration (Dehydr), and rectal temperature (Trectal), both at rest and after both 60 min of exercise in the four experimental conditions.

	Temperate				Heat			
	Euhydration (EU-T)		Dehydration (DE-T)		Euhydration (EU-H)		Dehydration (DE-H)	
	Rest	Exercise	Rest	Exercise	Rest	Exercise	Rest	Exercise
**USG**	1.020 ± 0.006	1.018 ± 0.009	1.027 ± 0.006	1.028 ± 0.005	1.019 ± 0.008	1.022 ± 0.008	1.026 ± 0.004	1.027 ± 0.004
**Hb (g/dL)**	15.6 ± 0.7	16.9 ± 1.1 *	16.1 ± 1.6	17.8 ± 1.2 *	15.9 ± 1.6	17.8 ± 1.4 *	15.6 ± 2.9	17.3 ± 3.1 *
**Htc (%)**	45.1 ± 1.1	46.2 ± 2.5	45.7 ± 3.2	46.8 ± 2.6	45.3 ± 2.6	47.2 ± 2.3 *	45.9 ± 2.8	47.2 ± 2.8 *
**PV (%)**	_	−9.4 ± 7.5	_	−11.4 ± 4.1	_	−13.6 ± 7.8	_	−12.3 ± 6.3
**BM (kg)**	80.1 ± 9.1	79.2 ± 9.1 *	79.4 ± 9.4	78.7 ± 9.5 *	79.9 ± 8.8	78.8 ± 8.8 *	79.4 ± 8.9	78.4 ± 8.9 *
**SR (L/h)**	_	0.83 ± 0.3	_	0.65 ± 0.2	_	1.2 ± 0.5 ^¥^	_	1.03 ± 0.43
**Dehydr (%)**	0 ± 0.6	1.04 ± 0.9 *	0.91 ± 0.8	1.75 ± 1.1 *	0.1 ± 0.7	1.6 ± 0.7 *	0.81 ± 1.2	2.10 + 1.2 *
**Trectal (°C)**	36.9 ± 0.4	37.6 ± 0.5 *	37.0 ± 0.3	37.8 ± 0.3 *	37.0 ± 0.4	38.0 ± 0.5 *	36.8 ± 0.2	38.0 ± 0.5 *

* Significantly different from rest (*p* < 0.05); ^¥^ Significantly different from HY-T (*p* < 0.05).

**Table 3:** Isolated effects of heat stress on reduced glutathione (GSH), oxidized glutathione (GSSG) and the GSH/GSSG ratio in plasma and erythrocytes.

	35 °C		41 °C	
	Plasma	Erythrocytes	Plasma	Erythrocytes
**GSH (µmol/L)**	0.0 ± 0.0	1.51 ± 1.46	0.04 ± 0.09	2.82 ± 1.14 *
**GSSG (µmol/L)**	0.17 ± 0.06	2.90 ± 0.78	0.17 ± 0.08	3.03 ± 0.83
**GSH/GSSG ratio**	0.0 ± 0.0	0.55 ± 0.52	0.19 ± 0.38	0.95 ± 0.34

* Significantly different from erythrocytes at 35 °C (*p* < 0.05).
